# Untangling the Knots of Regulatory T Cell Therapy in Solid Organ Transplantation

**DOI:** 10.3389/fimmu.2022.883855

**Published:** 2022-06-01

**Authors:** Gabriel Orozco, Meera Gupta, Roberto Gedaly, Francesc Marti

**Affiliations:** ^1^ Department of Surgery - Transplant Division, College of Medicine, University of Kentucky, Lexington, KY, United States; ^2^ Alliance Research Initiative [Treg cells to Induce Liver Tolerance (TILT) Alliance], University of Kentucky College of Medicine, Lexington, KY, United States; ^3^ Lucille Parker Markey Cancer Center, University of Kentucky, College of Medicine, Lexington, KY, United States

**Keywords:** regulatory T-cells, tolerance induction, transplantation, cellular therapy, adoptive therapies

## Abstract

Numerous preclinical studies have provided solid evidence supporting adoptive transfer of regulatory T cells (Tregs) to induce organ tolerance. As a result, there are 7 currently active Treg cell-based clinical trials in solid organ transplantation worldwide, all of which are early phase I or phase I/II trials. Although the results of these trials are optimistic and support both safety and feasibility, many experimental and clinical unanswered questions are slowing the progression of this new therapeutic alternative. In this review, we bring to the forefront the major challenges that Treg cell transplant investigators are currently facing, including the phenotypic and functional diversity of Treg cells, lineage stability, non-standardized *ex vivo* Treg cell manufacturing process, adequacy of administration route, inability of monitoring and tracking infused cells, and lack of biomarkers or validated surrogate endpoints of efficacy in clinical trials. With this plethora of interrogation marks, we are at a challenging and exciting crossroad where properly addressing these questions will determine the successful implementation of Treg cell-based immunotherapy in clinical transplantation.

## Introduction

Since the inception of transplant programs, the discovery and use of immunosuppressive drugs have played a critical role in preserving allograft function. After several decades of implementation, these immunosuppressive regimens have efficiently decreased the incidence of acute graft loss. However, long-term and chronic allograft rejection rates remain pervasive and, together with the severity of side effects in the allograft recipient population, makes the pursuit of therapeutic alternatives a medical necessity. A better understanding of self-tolerance mechanisms has facilitated different approaches aiming at rebalancing alloantigen-reactive conventional T-cells (Tconv) and immunosuppressive regulatory T cells (Tregs). This is a clear conceptual shift from the current standard multidrug-based protocols focused on halting effector immune responses.

CD25^hi^FoxP3^+^ Treg cells represent 1-5% of circulating CD4^+^ T lymphocytes and are essential in maintaining peripheral immune tolerance and homeostasis. After transplantation, the frequency of circulating Tregs in tolerant recipients is higher compared to patients with acute allograft rejection (1, 2). Increasing evidence also suggests that the balance between graft-reactive effector cells and graft-protective suppressor Tregs plays a role in organ engraftment and long-term allograft survival (3, 4).

Despite a decade of major progress in Treg research, technical limitations and significant gaps in our knowledge of Treg cell biology continue to hinder our ability to harness the therapeutic potential of these cells to induce allograft tolerance. This review summarizes achievements, current status and future challenges in the clinical implementation of Treg cell-based immunotherapy in solid organ transplant (SOT) recipients.

## Achievements and Current Status of Clinical Trials

The ability to isolate and expand Treg cells under good manufacturing practice (GMP)-compliant conditions paved the way for the clinical use of adoptive Treg cell transfer to induce allospecific tolerance in SOT patients. The first pilot study in SOT was reported by Todo et al. (5) in 10 liver transplant recipients using donor-specific Treg-enriched cell product in combination with standard immunosuppressive drugs that were gradually discontinued over a period of 18 months. All 10 recipients maintained stable graft function. Seven patients successfully achieved weaning of drugs between 16 and 33 months. All three patients who developed mild rejection during the immunosuppression weaning process underwent transplantation for autoimmune liver disease, which original autoimmune effector-regulatory unbalance may account for the difficult long-term control of effector responses. Since Todo’s report, five more original manuscripts have been published to date in SOT, four of them in kidney transplant patients and another in liver recipients (summarized in **Table 1**). Across all studies with at least one-year follow-up, fifty-four SOT recipients who received a single infusion of autologous Tregs had 100% survival, no episodes of graft loss, no increased risk of infection, and no report of *de novo* cancer (6–10). Only two patients suffered mild adverse events: one experienced mild and transient cytokine release syndrome (9), and another developed donor-specific antibodies one-year-post transplant and primary disease recurrence after a two-year follow-up (7). Furthermore, among 28 kidney transplant recipients receiving autologous transfer therapy with Tregs, the ONE study reported a significant decrease in the incidence of viral infections after transplant (12). Like the Todo et al. study, the stability of transplant function in Harden et al. study (10) also permitted minimization of immunosuppression, revealing a significant reduction of inflammatory cell populations in the transplanted organ as a result of Treg transfer. Overall, the published results support feasibility and safety of Treg infusion procedures in SOT patients and disclosed promising early data on feasibility of drug immunosuppressive minimization/discontinuation (**Table 1**). They are also uncovering multiple challenges that may harness the progression of immunotherapies in the clinic, including phenotypic and functional diversity of Treg cells, lineage stability, optimization of *ex vivo* Treg cell manufacturing process, adequacy of administration route, inability of monitoring and tracking infused cells, absence of organ specificity/trafficking markers in Treg cells, and lack of biomarkers or validated surrogate endpoints of efficacy in clinical trials. Importantly, measurements and report outcomes are often not comparable among different trials or centers, which makes it difficult to standardize methodologies and verify and validate data for consistency.

**Table 1:** Published studies evaluating Treg transfer therapy after solid organ transplantation.

Study	Clinical setting	Manufacturing process	Phenotype and purity of infused Treg cells	Administration and Tracking	Outcomes and safety
Todo et al. (5)	• Post-liver transplant patients. • 10 patients, 3-5 year follow up. • Weaning from immunosuppression 6 months after transplant	**Isolation:** No isolation.**Expansion**: 2-weeks co-culture recipient lymphocytes with irradiated donor cells with anti-CD80/CD86.**Preservation**: no preservation	Infused lymphocytes. 58.6% CD4^+^, 16.9% CD8^+^.Tregs represented 24.8% of infused CD4+ Tcells.	• Peripheral IV infusion. • No tracking.[Monitored differential lymphocytes counts in peripheral blood, including Tregs.]	• Cell infusion well tolerated by all recipients. • Seven patients successfully achieved uneventful weaning and completed cessation of immunosuppressive therapy. • Three patients showed acute cellular rejection symptoms during weaning
Chandran et al. (6)	• Three kidney transplant recipients. • Follow up biopsies at 2 weeks and 6 months after infusion. • Follow-up for one year after infusion.	**Isolation**: FACS sorting of CD4^+^ CD127^low/-^CD25^+^ from one unit of blood**Expansion**: 14-day culture with anti-CD3-CD28 paramagnetic beads, IL-2 and deuterated glucose (No Rapamycin).**Preservation**: none.	1x10^9^ Tregs with an average of 95% purity for FoxP3^+^ cells, >97% for CD4, and viability >99%. (Post-expansion)	• Peripheral IV infusion. • Tracking: Deuterated glucose.	• Cell infusion well tolerated by all recipients. • One patient developed (spontaneously resolved) leukopenia. • 100% patients and graft survival ater 1 year • Tregs circulating concentration peaked at one week. Deuterium signals detected up to 3 months after infusion ONLY in Treg cells
Mathew et al. (7)	• Nine kidney transplant recipients. • Three tiers of cell dosing (n = 3 per group): 0.5 × 10^9^, 1 × 10^9^, and 5 × 10^9^ Tregs/recipient. • Control group: historical cohort with identical immunosuppression	**Isolation:** Immunomagnetic isolation of CD4+, CD25+ cells from cryopreserved leukapheresis product.**Expansion:** 21-day culture with anti-CD3-CD28 paramagnetic beads, IL-2 TGFβ and Rapamycin.**Preservation :** Leukapheresis product collected one month before transplant.	>98% purity for CD4^+^ CD25^+^ cells and >80% for FoxP3^+^ FoxP3^+^ cells (Post-expansion)	• Peripheral IV infusion on postoperative day 60. • No tracking.[Monitored differential lymphocytes counts in peripheral blood, including Tregs.] • 5–20 fold increase of Tregs percentages in all Treg infusion recipients. Increase stable in most patients until the one-year mark.	• Cell infusion well tolerated by all recipients. • 100% patients and graft survival after 2 years. • Biopsy 3 months after cell infusion: no signs of rejection. Biopsy 1 year after cell infusion: one episode of subclinical rejection associated with immunosuppression non-compliance. • One subject with lowest Treg dose infusion developed donor-specific antibodies 1-year post-transplant. In the two-year follow-up, the patient developed primary disease recurrence.
Roemhild et al. (8)	• 11 kidney transplant recipients received an infusion of expanded autologous Tregs.Dosage design: three escalating doses: 0 (c), 0.5, 1, 2.5-3 x 10^6^ Treg/Kg (n = 3-4 patients/study group).All groups received drug immunosuppression Follow-up: three years.	**Isolation:** 2 step immunomagnetic isolation from 40-50 ml of peripheral blood:-1^st^: CD8 negative selection.-2^nd^: CD25 positive selection.**Expansion:** 21-day culture with anti-CD3-CD28 paramagnetic beads, IL-2 and Rapamycin.**Preservation:** none.	>1x10^9^ cells. 91.9% were CD4^+^ CD25^+^ FoxP3^+^. (Post-expansion)	Peripheral IV infusion • Tracking TCR repertoire Monitoring Tregs: • Tregs group: significant increase in Tregs counts and favorable Tregs/Teffector ratio for up to eight weeks after infusion. • Control group: Decreased Treg levels compared to baseline for up to 12 weeks after kidney transplantation.	• Cell infusion well tolerated by all recipients • 100% patients and graft survival after 2 years. • Treg therapy was significantly associated with successful weaning of drug therapy (p<0.001 at three years). • 10 patients in Tregs therapy were successfully weaned to low-dose tacrolimus monotherapy within 48 weeks. 2 patients required temporal or continuous reversal to triple immunosuppression therapy.
Sanchez-Fueyo et al. (9)	9 liver transplant recipients received an infusion of expanded autologous Tregs 3-16 months after transplant. Patients were assigned to one of two escalating doses: 1 x10^6^ Tregs/Kg (3pt) or 4.5 x10^6^ Tregs/Kg (6 pt).All patients received standard immunosuppression.Follow-up: 12 months.	**Isolation:** 2 step immunomagnetic isolation from 40-50 ml of peripheral blood:-1^st^: CD8 negative selection.-2^nd^: CD25 positive selection.**Expansion:** 36-day culture with anti-CD3-CD28 paramagnetic beads, IL-2 and Rapamycin.**Preservation:** Expanded Treg product	61-92% of cells were CD4^+^ CD25^+^ FoxP3^+^. Viability after thawing: 58-89% (Post-expansion).	• Peripheral IV infusion of expanded Treg cells thawed just before administration. • No tracking. • Monitoring: Six patients who received 4.5 x10^6^ Tregs/Kg had an increase in circulating Tregs noticeable from day 3 to 1 month. This increase was not observed in patients receiving 1 x10^6^ Tregs/Kg.	• No episodes of rejection during the follow-up period. • One patient receiving 4.5 x10^6^ Tregs/Kg developed mild temporary adverse effects.
Harden et al. (10, 11)	12 kidney transplant recipients received autologous Tregs infusion at post-operative d5Control group: 19 kidney transplant recipientsDosage design: 3 + 3 dose-escalation (three patients at each dose). Doses: 1, 3, 6 or 10 x 10^6^ Treg/KgAll patients received standard immunosuppression Follow up: four years	**Isolation:** immunomagnetic isolation. 2 steps:-1^st^: CD8 negative selection.-2^nd^: CD25 positive selection.**Expansion:** 36-day culture with anti-CD3-CD28 paramagnetic beads, IL-2 and Rapamycin.**Preservation:** Expanded Treg product	91.6% ± 9.3% of total cells were CD4+ CD25+ FoxP3+ After thawing, >70% of cells were CD4^+^ CD25^+^ FoxP3^+^. Viability after thawing: 58-89%. (Post-expansion)	• Peripheral IV infusion. Premedication with acetaminophen and antihistamine. Unfractionated heparin for 48 hours beginning on the day of infusion.No tracking. • Monitoring: Two weeks after transplant, observed dose-dependent increase in circulating number of Treg cells.	• Cell infusion: well tolerated by all recipients • 100% patients and graft survival after 2 years in all groups. • 100% patients and graft survival after 2 years in all groups. • Lower incidence of composite opportunistic infection with polyomavirus or cytomegalovirus in the Treg group. • Four patients in the Treg therapy cohort had successful minimization of immunosuppression (100% of patients attempted).

Tregs, regulatory T cells; FoxP3, forkhead box P3; IV, intravenous.

## Phenotypic Diversity

The efforts to characterize Tregs have revealed a broad spectrum of phenotypes in cells capable of engaging different suppressive mechanisms to control particular immune effector cell responses. The initial identification of these suppressor cells as CD4^+^ CD25^+^ T cells was substantiated by mouse experiments where their removal led to severe autoimmunity, which could be prevented after reconstituting these cells back to circulation (13, 14). In 2003, the forkhead box transcription factor FoxP3 was identified as an essential molecular marker of Treg cell development, differentiation and function. Since then, FoxP3 been considered as the defining Treg cell lineage “master-regulator” (15) and CD4^+^ CD25^+^ FoxP3^+^ as the distinctive core Treg phenotype. Expression of the interleukin-7 receptor (IL-7R) α chain (CD127) on the surface of Treg cells inversely correlates with FoxP3 expression and is another convenient marker for Treg cells as it provides an additional distinction between CD127^high^ (FoxP3^-/low^) and CD127^-/low^ (FoxP3^high^) subpopulations. In combination with CD25 during flow cytometry analysis, CD127 can be used as biomarker for analysis and, because of the expression on the cell membrane, for isolation of Tregs (16, 17).

Treg cells can be also categorized by the expression of another membrane marker, CD45RA. Consequently, functionally suppressive Treg population can be distinguished between naïve resting Tregs, with high proliferative potential (FoxP3^low^ CD25^low^ CD45RA^+^), and terminally differentiated, short-living Treg cells with low proliferative potential (FoxP3^high^ CD25^high^ CD45RA^-^) (18, 19). Accordingly, as proposed by Arroyo-Hornero et al. (20) and supported by Canavan et al. in Crohn’s disease patients (21), the segregation of the initial population of Treg cells based on the expression of CD45RA should be taken into consideration as CD45RA^+^ Tregs, but not CD45RA^-^, maintain a stable Treg signature after expansion. In a similar context, the expression of the Ikaros transcription factor family member, Helios, has been associated with lineage-committed, thymus-originated FoxP3^+^ Treg cell and, therefore, regarded as potential biomarker for therapeutic competent Treg cells. In mice, Helios^+^ and Helios^−^ Treg subpopulations are phenotypically and functionally distinct and express different TCR repertoires (22, 23). However, similar studies in human Tregs have not generated consistent results (24–27). A recent report by Lam et al. (28) suggests that Helios expression in Treg cells may be an important marker of lineage stability, although it does not have a direct role in the maintenance of the lineage-committed state. Co-expression of the surface markers T cell immunoreceptor with Ig and ITIM domains (TIGIT) and Fc Receptor-like 3 (FCRL3, CD307c) in Helios^+^ FoxP3 Treg cells (29) could facilitate reliable identification and selection of Helios^+^ Treg cells.

A shortcoming in the published literature of Treg cell-based clinical trials is the inconsistency and poor definition of the initial population of isolated Treg cells. Further phenotypic characterization is needed to identify the most appropriate population from which to expand FoxP3^+^ Tregs for use in adoptive transfer approaches, which will also help to determine whether a unique initial phenotypic Treg cell profile is well-suited to fulfill the specific demands of different target tissues.

## Lineage Stability

Lineage stability refers to the capability of a Treg cell to sustain immunosuppressive function in different environments and generate a progeny with similar characteristics after replication. The stable expression of signature genes for Treg cell lineage commitment should be considered as a critical parameter in the clinical competent population of Treg cells. Epigenetic changes such as DNA methylation, histone modification, and non-coding RNA synthesis regulate gene expression and cellular differentiation (30). Despite the phenotypic variability of Tregs, the epigenetic pattern can be used to identify a cell lineage with a stable immunosuppressive function. Indeed, the type of Treg-specific CpG hypomethylation pattern (TrHMP) is regarded as a more specific biomarker of functionally stable Treg than mere FoxP3 expression (31). The TrHMP includes hypomethylation of signature genes such as FoxP3, CTLA, GITR, and Helios (32), is heritable, independent of FoxP3 expression, and persists after TCR stimulation and in different culture conditions (30, 32). TrHMP is also linked to the suppressive strength of Treg cells as observed with *in vitro* induced (iTregs) cells. Despite the expression of FoxP3, iTregs show a TrHMP similar to activated Tconv, are less suppressive and demonstrate less lineage commitment than natural, thymus-originated Tregs (nTregs) (32). The strong association between Treg lineage stability and specific epigenetic imprinting supports the use of TrHMP as biomarker for Treg lineage determination/stability and the inclusion among the most reliable parameters currently available as criterion for the identification of functional Treg cells in experimental settings. However, the methylation status pattern is not included in any reported clinical study as a release criterion for clinically competent Treg cells (**Table 1**). In zaddition, the capacity to track lineage stability of infused Tregs *in vivo* in the clinic is limited (7). In Chandran et al. study, the authors transferred deuterium-labeled Tregs into kidney transplant recipients. The fact that deuterium signals were only detected in the Treg population within three months post-infusion suggests the lineage stability of infused cells (6).

## Manufacturing Process

While the Treg ability to inhibit the effector immune reactions that trigger graft rejection has been demonstrated in numerous pre-clinical studies, their low concentration in peripheral blood has become a major obstacle to their clinical application (33). However, refinements in the manufacturing process under Good Manufacturing Practices (GMP)-compatible conditions now facilitate escalating the cellular yield up to 2,000-fold (8). This process includes three main steps: isolation, expansion, and preservation.

### Isolation

The two most common methods for Treg isolation are Fluorescence-Activated Cell Sorting (FACS) and immuno-magnetic cell separation. FACS has been primarily used for research and analytical purposes, but recent adaptations to comply with GMP legislation have allowed its clinical use. FACS can distinguish very specific cellular subpopulations, sort cells based on the degree of expression of particular markers and discriminate several subpopulations simultaneously. However, FACS-based isolation of Treg cells for human therapy relies only on extracellular markers to identify the target population (34). Another technical limitation of this method is that the sorting efficiency is reduced when the population of interest is rare, requiring lengthy processing times from a large initial cell population (35). Still, some groups have successfully isolated Treg cells for clinical interventions with FACS (6, 36, 37), and the progress towards using more complex membrane marker combinations to define the initial Treg population may help a broader use of this technology as cell isolation procedure.

Immunomagnetic cell separation is the current method of choice for Treg isolation in clinical trials. Biotechnology companies have developed closed, automatic systems to comply with GMP regulations. In this method, magnetized particles are conjugated with antibodies, and consecutive steps of negative and positive selection allow the isolation of a specific Treg cell population. The purity of the isolation can increase by selecting multiple markers during a single pass of negative selection (e.g., CD8 and CD19) (11, 38). Most published Treg-based clinical trials in SOT reported the use of magnetic immunoselection isolation technique as a two-step procedure with initial CD8 depletion and subsequent CD25 enrichment (7–10). A significant loss of targeted cells after each selection step, the necessity of fine-tune optimization to find the optimal balance between cell yield and purity, the lack of discriminatory capacity between low or high expression of cellular markers, and the elevated cost of the procedure (specific equipment and supplies) are some of the shortcomings associated with Treg isolation by magnetic immunoselection. As such, more versatile GMP technologies are needed to improve yield and purity of clinical-grade quality Treg cell isolates and facilitate the standard implementation of this technology in clinical practice.

### Expansion

Tregs constitute 1-5% of the total circulating CD4^+^ T lymphocyte population (39). These low numbers and favoring cell purity over yield in the isolation process make *ex vivo* expansion a critical step towards successful cell therapy implementation. The main strategy for *ex vivo* expansion is establishing cell culture conditions to preferentially activate and expand Treg cells while preventing the replication of other potential contaminant cell types. Expansion protocols can produce up to 2,000-fold amplification of Treg cell numbers (36) and are based on the concomitant engagement of the T cell receptor (TCR) and the costimulatory receptor CD28, and high doses of the T cell growth and survival factor IL-2. Addition of mTOR inhibitors (e.g., rapamycin, everolimus) promotes the selective expansion and suppressive activity of Tregs (40, 41) while preventing Tconv activation and growth. Mechanistic evidence supports that mTOR signaling pathway is a critical regulator of effector Tconv homeostasis and function but not of Tregs (42–44). In fact, PI3K/Akt/mTOR activation represses Treg differentiation, and the inhibition of the Akt pathway is crucial to promote the activation of FoxP3 (45–47). Metabolically, Tconv depends on the mTOR-driven glycolytic pathway for a rapid supply of energy and molecular precursors (48); in contrast, the energy demand of Treg cells is fulfilled by the constant crosstalk between glycolytic and oxidative mitochondrial metabolic arms (49–51).

Prolonged stimulation of Tregs triggers epigenetic changes leading to suboptimal TCR signaling and progressive hypermethylation of Treg-specific demethylated regions (52). These epigenetic alterations can change the quality of the final cell product by promoting Treg conversion to Tconv or reducing their suppressive function (52, 53). Upon activation, Tregs may undergo a progressive shift from CD45RA^+^ to CD45RA^-^ phenotype (54). Upon further expansion, the CD45RA^-^ fraction experiences a decline in both FoxP3 expression and suppressive activity (54). As mentioned, adding an mTOR inhibitor such as rapamycin sustains the expansion and suppressive activity of Tregs (40), but also induces the conversion of conventional CD4^+^ T cells into iTregs. However, these iTregs do not possess the TrHMP hypomethylated signature of Treg genes and can revert into non-suppressive cells in the absence of rapamycin. Therefore, as suggested by Battaglia et al. (40), careful attention and appropriate quality controls must be in place when mTOR inhibitors are included in the expansion protocol for Treg cell therapy. For clinical application, the initiation of the expansion phase with highly purified and well-defined population of Treg cells seems the appropriate strategy. Overall, these studies highlight the importance of optimizing cell culture conditions (composition and duration) and quality control assessments in the expansion protocols for Treg manufacturing (52, 54). The progress of Treg immunotherapy demands establishing relevant mechanistic links between pre- and post-expansion phenotype, suppressor function and epigenetic profile of Treg cell populations with corresponding clinically relevant outcomes of operational tolerance or reduced rejection.

### Preservation

For far-reaching applications of Treg cell-based therapy, it is essential to ensure the stability of the cell product during storage, including optimal cell viability, recovery and functionality. Widening the window between the collection and application of Tregs for adoptive therapy would increase the flexibility of their clinical use. Because preservation techniques can potentially change the yield, viability, and activity of Tregs, they are considered a new therapeutic biological product from a regulatory point of view (55).

Tregs can be cryopreserved before isolation (as peripheral blood mononuclear cells, PBMCs), just after isolation, or after the expansion phase. Treg cell recovery rates from cryopreserved PBMCs fluctuate between 35 to 63% (55–61). Using isolated Tregs cryopreserved in liquid nitrogen for up to one year, Peters et al. reported a viability of 70-80%, with a suppressive capacity that was significantly impaired after thawing but recovered after activation (38). Kaiser et al. found better recovery rates and cellular viability by using cryogenic solution of 5% DMSO instead of 10% DMSO (55). Cryopreserved Tregs after three or four cycles of re-stimulation did not alter their original phenotype or suppressive function (56). Different groups have reported using cryopreserved Treg cells in SOT patients. Mathew et al. cryopreserved the leukapheresis product approximately one month before kidney transplant, their expansion protocol lasted 21 days, and the infusion of Tregs was given 60 days after surgery (7). Harden et al. and Sanchez-Fueyo et al. cryopreserved the Treg product after isolation and expansion and thawing was performed at the bedside of the patient prior to administration (9, 10). Fraser et al. reported the feasibility of infusing pre-expanded cryopreserved Tregs, showing a reported cell recovery >90%, viability >75% and suppressive function of >80% (11).

Using fresh starting material may have recognized advantages, but the ability to cryopreserve also allows for a more flexible, convenient, efficient -and less expensive- manufacturing process that can be easily managed and scheduled in cellular therapy laboratories (9, 10). However, the effect of cryopreservation on Treg phenotypic and functional parameters and on subsequent clinical outcomes has to be properly established. Such assessments would have a profound logistical impact on clinical trial design, infusion timelines and testing requirements for future studies.

## Administration Route

Too often, the cell delivery method is an overlooked factor that may have a direct effect on treatment bioavailability to the target organ and, as such, a determining factor for assessments of feasibility, safety and efficacy outcomes of treatment (62, 63). There are two principal methods to introduce cells into the body: systemic delivery and local delivery into the organ. The most common method for Treg cell infusion is systemic intravenous (IV) injection. IV injection allows for wide distribution of cells throughout the body, and it has the advantage of being minimally invasive with low/minimal safety risks in early phase clinical studies. With this methodology, there are several hurdles to overcome in order to deliver cells to the target organ and have them engrafted. IV delivered cells have to pass through the lungs before they can distribute throughout the body. This pulmonary “first-pass” effect results in significant entrapment of cells (64) caused by the estimated size of Tregs (10–15 μm diameter) (65–68), as observed with microsphere particles of this size (64, 69). Similarly, clinical studies with IV-delivered stem cell infusion showed that the majority of cells get trapped in the lungs after intravenous administration (64, 69, 70). Likewise, systemic infusion of expanded tumor infiltrating lymphocytes (TILs) resulted in higher concentrations of cells in lung, liver, and spleen (71).

The optimal method of therapeutic cell delivery will always depend on the mechanism of action of the cell product. Since Tregs cannot exert their organ-protective effect distally, the delivery system must reach the target organ or allow Treg cells to migrate toward it. The alternative to systemic infusion is the direct local delivery into the organ. This approach can provide a high concentration of Tregs in a first passage where all injected cells have opportunity to interact with post-capillary endothelia of the target organ (72). Direct intra-arterial infusion of stem cells into the brain has proven to significantly enhance cellular engraftment and concentration in animal models of brain ischemia when compared to systemic IV administration (73–75). Also reported, infusion of radiolabeled TILs in the hepatic artery is followed by a rapid increase and slow decline in the intensity signal of the liver (76). However, a disadvantage of local injection is that it may cause further local damage in tissue that, such as a SOT, is already particularly sensitive. It has also been shown that, although direct injection increased localization, it did not necessarily increase engraftment or survival (77). Animal models using direct intra-arterial delivery of mesenchymal stromal cells to the kidney have shown retention of cells in the renal cortex (78) and induction of a favorable tolerogenic milieu after transplantation (79–85). To the best of our knowledge, all currently active clinical protocols using adoptive transferred Treg cells in transplantation are using systemic IV delivery of cells. As safety is the necessary focus of these phase I/(II) studies, alternative routes of cell administration have become an understudied area that remains to be properly addressed. Developing efficient cell delivery protocols could significantly improve the effective implementation and outcomes of Treg-based cell therapy in SOT.

## Monitoring and Tracking Infused Cells

Regardless of the infusion route, the success of any cell-based immunotherapy relies on efficacy of cell trafficking and recruitment to the targeted area where they must remain functional. Tracking these adoptive cells *in vivo* becomes critical to evaluate their delivery, biodistribution and therapeutic response. However, our ability to longitudinally interrogate the migration and fate of infused Treg cells throughout the body remains elusive. In fact, it has become one of the most challenging limitations in current Treg cell immunotherapy studies.

There are only a few studies reporting the *in vivo* assessment of the distribution and fate of infused Treg cells in humans. Oo et al. used single-photon emission computed tomography (SPECT) to track the distribution of autologous Tregs marked with ^111^Indium tropolonate (^111^In) in four patients with autoimmune hepatitis. At 24 hours, they detected a predominant distribution within the liver (22-44%), spleen (11-24%), and bone marrow (9-13%). Tregs persisted in the liver for 72 hours until the ^111^In was no longer detectable (86). Bluestone et al. used non-radioactive labeling of deoxyribose with deuterium for tracking Treg cells after infusion in type-1 diabetes patients (37). They observed a peak concentration of circulating Tregs between days 7 and 14 with a subsequent decline. Ninety days later, the concentration was 25% of the maximum, and one year after, labeled Tregs were still detected. They reported an initial fast decay phase of infused cells with a half-life of 19.6 days, followed by a slower decay phase. Chandran et al. demonstrated similar kinetic and stability pattern: Tregs peaked in the first week, had a bi-phasic decay, and were still detectable circulating one month after infusion, but were undetectable at the 3-month mark (6). However, a study in non-human primates reported strikingly different results: using carboxyfluorescein succinimidyl ester (CFSE)-labeled cells, Singh et al. observed a rapid decrease of Tregs in peripheral blood during the first three days after infusion, and were barely detectable after 16 days. The uptake and clearance of infused Tregs in bone marrow and lymph nodes followed a similar pattern as with concentrations in blood. They also reported a significant change in phenotype, with less than 30% of CFSE-labeled cells holding the CD25^+^FoxP3^+^ phenotype by day 16 (87). Although cell manufacturing and labeling protocols differed among studies, and accounting for possible inter-species variability, the inconsistent results among available studies underscore current limitations to assess *in vivo* trafficking, homing and fate of infused Treg cells to the transplanted organ.

Novel approaches for monitoring the biodistribution and organ trafficking efficacy of adoptive Treg cells after infusion are in dire need. In the absence of standard non-invasive modalities to assess treatment responses, allograft biopsy analyses of FoxP3 mRNA expression in the transplanted organ, either alone or as a ratio with *GranzymeB*, are used as surrogate markers for infiltrated Treg and Teff cells, respectively (88–101). New non-invasive imaging technologies such as SPECT, Positron Emission Tomography (PET), Magnetic Resonance Imaging (MRI) or hybrid modalities such as MRI-SPECT in combination with computational biology (102–104) still require validation and standardization. However, they are among emerging technologies that, once implemented into clinical practice, will significantly help improve the efficacy of current cell-based therapy protocols.

## Antigen-Specific Treg Cells

The generation of antigen-specific Treg cells is a valuable new approach to provide local, more restricted, immune tolerance (105–109) with cells that are efficiently trafficking to tissues that express cognate antigens (110, 111). Efforts to generate antigen-specific Tregs are currently focused on two different strategies: *ex-vivo* induction of Tregs by stimulation of antigen-directed CD4^+^ effector Tconv cells (112), and engineering synthetic T-cell receptors (TCRs) or chimeric antigen receptors (CARs) with target-tissue specificity (113). The reported lineage instability of iTregs cells under inflammatory conditions precludes the clinical use of these cells. To potentially overcome the limitations to generate clinically efficient antigen-directed iTreg cells, gene-editing or transgenic approaches are being applied to induce stable expression of FoxP3 or other Treg signature proteins, as well as to identify key gene targets and pathways involved in the regulation of Treg function and stability (112, 114–118).

On the other hand, the genetic introduction of engineered TCRs and CARs can provide antigen-specificity to polyclonal Tregs (106, 111, 113, 119–121). The ectopic expression of TCRs in Treg cells allows the targeting of processed intracellular antigens presented by HLA molecules. Several pre-clinical studies have demonstrated translational potential of this approach (120, 122–125). However, the HLA-restricted physiological activation limits the application of engineered TCRs and may acquire harmful specificities when mispaired with endogenous TCRs. Interestingly, enforcing the expression of MHC-I-restricted TCRs or not functional low affinity Tconv TCRs (126), enable human Treg cells to bypass the MHC requirement for antigen recognition. Also, instead of using exogenous TCRs isolated from Tconv cells, there is the option of using specific Treg TCRs, which have shown some structural differences (127–129). Another strategy may entail the creation of universal Treg donor cell lines by sequential genetic modifications of MHC molecules (130).

CARs are modular artificial receptors that combine an extracellular antigen-recognition domain and intracellular signaling and costimulatory domains. CAR-engineered effector T cells are being used to reprogram effector Tconv to target tumor cells in patients with blood cancers (131–134). The major advantage of CARs is their ability to recognize whole proteins expressed in target tissues unrestricted to MHC class I or II presentation. Therefore, unlike TCR-modified Treg, CAR-Tregs cells could be applicable to a larger number of patients. However, the design of the CAR should consider specific traits of the host Treg cell, such as the determination of optimal specificity and affinity/avidity of antigen recognition and identification of co-stimulatory signaling domains and accessory molecules that enhance suppressive activity without jeopardizing Treg lineage stability. Recent in-depth reviews comprehensively discuss current status and future prospects of engineered TCR- and CAR-Treg cells in different clinical settings (135, 136).

Currently, a multi-center clinical trial is investigating safety and tolerability of CAR-Treg therapy in HLA-A2 mismatched kidney transplant recipients (NCT04817774) (**Table 2**). These advanced genetic technologies in cell therapy should be implemented in clinical settings with restricted safety precautions and quality control assessments. Among the latter, the complex nature of Treg functional fitness needs careful attention to any TCR genetic manipulation as the maintenance of Treg identity depends on a fine-tuned strength of antigen-specific stimulation (113, 137, 138).

**Table 2:** Clinical trials evaluating Treg transfer therapy after solid organ transplantation*.

Study ID (Phase)	Treg product	Clinical settings
**NCT04817774 ** (I/II)	Antigen-specific CAR-Tregs (TX200-TR101)	Population: HLA-A2 mismatched living kidney donor transplant recipients.
	Dose: not specified.	Intervention: IV infusion of autologous CAR-Tregs.
	Three single ascending dose cohorts and an additional expansion cohort.	Follow-up: 84 weeks after infusion.
**NCT03943238 ** (I)	Autologous, polyclonal, *ex-vivo* expanded Tregs.	Population: Kidney transplant recipients.
	Dose: starts at 25x10^6^ cells.	Intervention: Two weeks after transplant, on separate days, IV infusion of:
	Escalated doses of Tregs if the donor chimerism is less than 25% after 60 days.	- Purified CD34^+^ and T cells from the kidney donor.
		- Autologous Treg cells.
		Follow-up: 2 years
**NCT03867617** (I/II)	Autologous, polyclonal, *ex-vivo* expanded Tregs.	Population: HLA-mismatched living donor kidney transplant recipients.
	Dose (cells/kg): Target dose: 1x10^7^	Intervention: IV infusion of autologous regulatory T cells + donor bone marrow + Tocilizumab.
	Dose range: 0.3-1.5 x10^7^	Follow-up: one year
**NCT03284242** (I)	Autologous, polyclonal, *ex-vivo* expanded Tregs.	Population: Kidney transplant recipients
	Dose: 50-300x10^6^	Intervention: IV infusion of Treg cells
		Follow-up: 2 years
**NCT02711826 ** (I/II)	Autologous, polyclonal, *ex-vivo* expanded Tregs.	Population: Kidney transplant recipients
	Dose: 100-1000 x10^6^ cells.	Intervention: IV infusion of autologous regulatory T cells 3-7 months after transplant.
		Follow-up: 405 days
**NCT03577431 ** (I/II)	Donor alloantigen-specific autologous Tregs.	Population: Liver transplant recipients.
	Dose: Target dose: 2.5 x 10 ^6^ cells.	Intervention: IV infusion of Treg cells.
	Dose range: 1-125 x 10^6^ cells.	Follow up: Until completion of study (At least 104 weeks, up to 4.5 years).
	Intent to treat analysis: 1-2.5 x 10^6^cells.	
**NCT03654040** (I/II)	Donor alloantigen-specific autologous Tregs.	Population: Liver transplant recipients.
	Dose: Target dose: 90x10^6^ cells.	Intervention: IV infusion of Treg cells.
		Follow up: Until completion of study (At least 104 weeks, up to 4.5 years).
**NCT02474199** (I/II) (completed)	Donor alloantigen-specific autologous Treg cells.	Population: Liver transplant recipients (2 to 7 years post Tx)
	Dose: 400 x 10^6^ cells (300-500 x 10^6^)	Intervention: IV infusion of Treg cells.
		Follow-up: 52 weeks.
**NCT02188719** (I) (Terminated)	Donor alloantigen-specific autologous Treg cells.	Population: Liver transplant recipients
	Dose: Cohort #1: No cells; #2: 25-60 million cells (target: 50 million); #3: 100-240 million cells (target: 200 million); #4: 400-960 million (target: 800 million).	Intervention: IV infusion of Treg cells.
		Follow up: 40 weeks after transplant.
**NCT02091232**(I) (completed)** (12)	Autologous, donor antigen reactive, *ex-vivo* expanded Tregs, stimulated with kidney donor PBMC in the presence of beltacept	Population: Kidney transplant recipients
		Intervention: IV infusion of Treg cells.
		Follow-up: 2 years

Tregs, regulatory T cells; CAR-Tregs, chimeric antigen receptor Treg; HLA, human leukocyte antigens; IV, intravenous; ALT, Alanine amino-transferase; GGT, Gamma-Glutamyl transpeptidase. *Clinical trials with unknown status are not reported in the table. **Results are published as part of the ONE study.

## Efficacy: Endpoints and Biomarkers

Traditional primary endpoints for treatment efficacy in transplant clinical trials include graft survival, death with a functioning graft, and quality of life (QoL). These ‘patient-centered’ endpoints are commonly evaluated by surrogate endpoints which, by definition, require adequate validation and should demonstrate robust ability to predict meaningful benefits. Effective use of surrogate endpoints offers the promise of more efficient assessment by providing earlier answers to questions of therapeutic efficacy. Common surrogate endpoints to assess transplant allograft survival include: subclinical, acute cellular, antibody-mediated and steroid-resistant rejection episodes.

The use of biomarkers as surrogates has facilitated the assessment of treatment efficacy in numerous clinical studies. Currently, there is no validated biomarker for treatment efficacy in organ transplantation, which is a fundamental limitation in clinical studies. QoL is a complex endpoint difficult to evaluate because it includes multiple physical, emotional and intellectual parameters that are subjective in nature (139). Although Treg transfer therapy studies are early phase I/II safety and feasibility trials, the evaluation of clinical efficacy is still a critical unresolved issue. To aggravate this limitation, current good short-term outcomes of standard drug immunosuppression regimens demand long-term evaluation of large cohorts to assess the efficacy of any novel therapeutic intervention (140, 141). Implementation of standardized measurable outcomes of direct relevance to patients (including graft function and QoL) is an obvious shortcoming of current SOT clinical trials. Multicentric trials such as *Treg Adoptive Therapy for Subclinical Inflammation in Kidney Transplantation* (TASK), *TReg Adoptive Cell Therapy* (TRACT), or the *ONE Study* are positive initial attempts to unify criteria of cell manufacturing and evaluation of SOT Treg-based clinical studies. However, current protocols still vary in essential criteria and further efforts are required to develop common designs in future clinical trials.

## Conclusion

The indispensable role of Treg cells to immune homeostasis and sustaining self-tolerance has awakened an exciting field of research in SOT. Aiming at improving the QoL and long-term outcomes of transplant recipients, Treg cell therapy appears as an attractive alternative to current standard immunosuppressive treatments. Recent bench-to-bedside progress is paving the way towards the successful application of cellular therapies to achieve transplant tolerance. We hope this review will help the reader appreciate the enormous therapeutic potential and also the challenges of Treg cell-based immunotherapy in transplantation.

## Author Contributions

Conception and design of the study, RG and FM. GO, RG, and FM wrote the review. MG provided significant revisions to the manuscript. All authors contributed to the article and approved the submitted version.

## Funding

This work was supported by the Treg Cells to Induce Liver Tolerance (TILT) Alliance Research Initiative of the College of Medicine at the University of Kentucky.

## Conflict of Interest

The authors declare that the research was conducted in the absence of any commercial or financial relationships that could be construed as a potential conflict of interest.

## Publisher’s Note

All claims expressed in this article are solely those of the authors and do not necessarily represent those of their affiliated organizations, or those of the publisher, the editors and the reviewers. Any product that may be evaluated in this article, or claim that may be made by its manufacturer, is not guaranteed or endorsed by the publisher.
